# Emerging Mechanistic Insights and Therapeutic Strategies for Pulmonary Arterial Hypertension: A Focus on Right Ventricular Dysfunction and Novel Treatment Pathways

**DOI:** 10.3390/biomedicines13030600

**Published:** 2025-03-01

**Authors:** Masab Mansoor, Andrew F. Ibrahim

**Affiliations:** 1 Edward Via College of Osteopathic Medicine–Louisiana Campus, 4408 Bon Aire Dr, Monroe, LA 71203, USA; 2 Texas Tech University Health Sciences Center School of Medicine, Lubbock, TX 79430, USA; andrew.ibrahim@ttuhsc.edu

**Keywords:** pulmonary arterial hypertension, vascular remodeling, right ventricular dysfunction

## Abstract

**Background/Objectives**: Pulmonary arterial hypertension (PAH) is a progressive vascular disorder characterized by increased pulmonary vascular resistance, right ventricular dysfunction, and high mortality rates. Despite advancements in vasodilatory therapies, PAH remains a life-threatening condition with limited curative options. This review aimed to explore emerging molecular mechanisms, novel therapeutic targets, and future research directions in PAH treatment, focusing on strategies to improve long-term patient outcomes. **Methods**: This review synthesized recent advancements in PAH pathophysiology and therapeutic development. A structured literature search was conducted on PubMed and ClinicalTrials.gov using keywords such as “Pulmonary Arterial Hypertension”, “vascular remodeling”, “metabolic dysfunction”, and “emerging therapies”. Studies published between 2015 and 2025 were included, with a focus on preclinical models, clinical trials, and translational research. Key areas of investigation include vascular remodeling, metabolic dysregulation, inflammation, and right ventricular dysfunction. The review also evaluated the potential of novel pharmacological agents, gene-based therapies, and AI-driven diagnostics for PAH management. **Results**: Recent studies highlight dysregulated BMPR2 signaling, epigenetic modifications, and inflammatory cytokine pathways as critical contributors to PAH progression. Emerging therapies such as JAK-STAT inhibitors, metabolic reprogramming agents, and mesenchymal stromal cell-derived extracellular vesicles (EVs) show promise in preclinical and early clinical trials. Additionally, AI-enhanced imaging and non-invasive biomarkers are improving PAH diagnostics. Future research directions emphasize precision medicine approaches and the development of RV-targeted therapies. **Conclusions**: PAH remains a complex and fatal disease requiring multifaceted therapeutic strategies beyond traditional vasodilation. Advances in molecular-targeted treatments, AI-driven diagnostics, and personalized medicine offer new hope for disease-modifying interventions. Future research must bridge translational gaps to bring novel therapies from bench to bedside, improving survival and quality of life in PAH patients.

## 1. Background

Pulmonary arterial hypertension (PAH) is a progressive disorder characterized by increased pulmonary vascular resistance, ultimately leading to right heart failure and premature death. Despite advancements in pharmacotherapy over the past two decades, PAH remains a life-threatening disease with limited curative options. Traditional treatments target endothelial dysfunction through pathways involving prostacyclin, endothelin, and nitric oxide (NO) signaling. However, these therapies primarily address vascular tone and do not sufficiently alter the disease course or prevent right ventricular (RV) failure, which is a critical determinant of prognosis in PAH patients [1].

### 1.1. Classification of Pulmonary Hypertension

**Figure 1:**
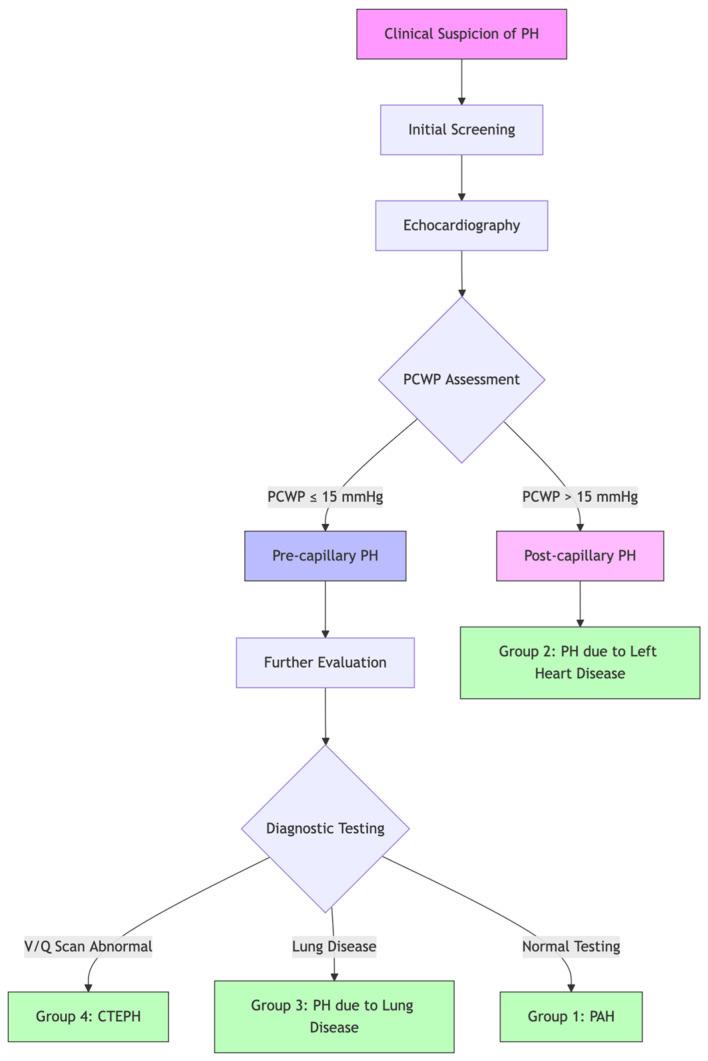
Diagnostic algorithm for pulmonary hypertension. This flowchart illustrates the systematic approach to diagnosing different types of pulmonary hypertension. Initial screening with echocardiography leads to pulmonary capillary wedge pressure (PCWP) assessment, which helps differentiate between pre- and post-capillary PH. Further diagnostic testing, including V/Q scan and evaluation for lung disease, guides classification into specific PH groups. PCWP = pulmonary capillary wedge pressure; CTEPH = chronic thromboembolic pulmonary hypertension; PAH = pulmonary arterial hypertension.

Pulmonary hypertension (PH) encompasses a spectrum of diseases categorized into five distinct groups based on etiology, pathophysiology, and treatment responses [2]. Understanding these subtypes is crucial for accurate diagnosis and appropriate management strategies. These classifications are displayed on Table 1. Figure 1 displays a diagnostic algorithim for PAH.

#### 1.1.1. Group 1: Pulmonary Arterial Hypertension (PAH)

PAH Includes idiopathic, heritable, drug-induced, and connective tissue disease-associated PAH and is characterized by endothelial dysfunction, excessive pulmonary vasoconstriction, and vascular remodeling. Standard therapies include prostacyclin analogs, endothelin receptor antagonists, and phosphodiesterase-5 inhibitors [3].

#### 1.1.2. Group 2: PH Due to Left Heart Disease

PH due to left heart disease is the most common form of PH, often secondary to heart failure with preserved or reduced ejection fraction [4]. Increased left atrial pressure leads to passive pulmonary congestion. Unlike PAH, vasodilatory therapies are generally contraindicated.

#### 1.1.3. Group 3: PH Associated with Lung Diseases and/or Hypoxia

PH associated with lung diseases occurs in chronic obstructive pulmonary disease (COPD), interstitial lung disease, and sleep-disordered breathing [5]. Hypoxia-induced vasoconstriction plays a central role. Treatment focuses on optimizing lung disease and oxygen therapy.

#### 1.1.4. Group 4: Chronic Thromboembolic Pulmonary Hypertension (CTEPH)

Chronic Thromboembolic Pulmonary Hypertension is caused by unresolved pulmonary emboli leading to vascular obstruction and remodeling [6]. Unlike PAH, it may be curable with pulmonary endarterectomy or balloon pulmonary angioplasty. Targeted medical therapy includes riociguat [7,8].

#### 1.1.5. Group 5: PH with Unclear/Multifactorial Mechanisms

PH with unclear multifactorial mechanisms includes hematologic disorders (e.g., sickle cell disease), metabolic disorders, and systemic conditions such as sarcoidosis [9]. Pathophysiology varies widely, requiring individualized management.

### 1.2. Recent Developments in PAH

Recent research efforts have focused on understanding the cellular and molecular underpinnings of PAH, highlighting novel pathogenic mechanisms, including inflammation, metabolic dysregulation, and genetic susceptibility. The role of oxidative stress and mitochondrial dysfunction in pulmonary vascular remodeling is now well recognized, with studies suggesting that therapies targeting these pathways may offer new therapeutic benefits [10]. Additionally, the involvement of the bone morphogenetic protein receptor type 2 (BMPR2) signaling in pulmonary vascular homeostasis has spurred interest in exploring its downstream effectors as potential drug targets [11].

Epigenetic modifications, such as DNA methylation and histone acetylation, have also been implicated in the pathogenesis of PAH. These alterations contribute to the sustained proliferation of pulmonary arterial smooth muscle cells (PASMCs) and endothelial dysfunction, promoting vascular remodeling and increased pulmonary pressure [12]. The concept of personalized medicine has gained traction, with studies suggesting that patient-specific transcriptional signatures could guide tailored therapeutic interventions [13].

Given the pivotal role of the right ventricle in PAH prognoses, recent efforts have sought to develop RV-directed therapies. Strategies include metabolic modulation, antifibrotic agents, and inotropic therapies aimed at preserving RV function and improving survival outcomes [14]. Furthermore, the potential for drug repurposing has gained interest, with compounds such as celastrol showing promise in ameliorating hypoxia-induced pulmonary hypertension through modulation of the phosphodiesterase 5 (PDE5)-cGMP-PKG signaling pathway [15].

This review aimed to provide an updated synthesis of emerging research on PAH, with a specific focus on novel molecular targets, epigenetic mechanisms, and innovative therapeutic approaches. By integrating insights from recent studies, we sought to highlight new avenues for improving patient outcomes and addressing the persistent challenges in PAH management.

## 2. Introduction

Pulmonary arterial hypertension (PAH) is a progressive vascular disease characterized by increased pulmonary arterial pressure, leading to right heart failure and high mortality rates. Despite advancements in pharmacotherapy, PAH remains a debilitating condition with limited curative options. Current treatment approaches, including prostacyclin analogs, endothelin receptor antagonists, and phosphodiesterase-5 inhibitors, primarily focus on restoring vascular tone but do not adequately prevent disease progression or right ventricular (RV) failure, which remains the leading cause of mortality in PAH patients [16].

### 2.1. Gaps in Current Research and Treatment

While significant strides have been made in understanding the molecular mechanisms underlying pulmonary arterial hypertension (PAH, Group 1), different forms of pulmonary hypertension (PH) have distinct pathophysiologies that impact diagnosis and treatment [17,18]. For example, PH due to left heart disease (Group 2) is driven by post-capillary pressure elevations from left ventricular dysfunction, making the use of vasodilators, a mainstay in PAH treatment, potentially harmful [19,20]. Similarly, PH due to lung diseases/hypoxia (Group 3) is predominantly caused by hypoxic vasoconstriction and vascular remodeling, requiring interventions focused on treating the underlying lung disease rather than targeting the pulmonary vasculature directly [21].

The diagnostic approach also varies among PH groups:

- Right heart catheterization (RHC) remains the gold standard for distinguishing PAH from other PH types by measuring pulmonary arterial pressures and pulmonary capillary wedge pressure (PCWP) [22];
- Echocardiography is often the initial screening tool due to its advantages of cost-effectiveness, wide availability, and safety [23];
- Ventilation–perfusion (V/Q) scans and CT angiography are critical in identifying chronic thromboembolic pulmonary hypertension (CTEPH, Group 4) [24].

Given these differences, a precise diagnosis is essential to avoid mismanagement, particularly when considering PAH-specific therapies such as endothelin receptor antagonists, prostacyclin analogs, and phosphodiesterase-5 inhibitors, which are only appropriate for Group 1 PAH [25]. Current research highlights the importance of epigenetics, inflammatory signaling, and metabolic dysfunction in PAH progression [26]. Additionally, the role of right ventricular dysfunction as a prognostic marker and therapeutic target remains an evolving field [27].

### 2.2. Objective of This Review

The aims of this review are the following:

1. Explore emerging molecular pathways involved in PAH pathogenesis;
2. Discuss novel therapeutic strategies, including epigenetic interventions, targeted metabolic therapies, and right ventricle-specific treatments;
3. Evaluate the potential for drug repurposing and future directions in PAH treatment.

By consolidating recent advancements, this review seeks to provide clinicians and researchers with updated insights into novel diagnostic and therapeutic strategies that could improve long-term outcomes for PAH patients.

## 3. Pathophysiology and Molecular Mechanisms of PAH

Pulmonary arterial hypertension (PAH) is a progressive vascular disease characterized by increased pulmonary vascular resistance (PVR), endothelial dysfunction, and excessive proliferation of pulmonary arterial smooth muscle cells (PASMCs), ultimately leading to right ventricular (RV) failure. Recent research has highlighted the complex interplay between genetic, inflammatory, and metabolic pathways contributing to PAH pathogenesis [28]. This section explores the cellular and molecular mechanisms underlying PAH development.

### 3.1. Endothelial Dysfunction and Vascular Remodeling

A hallmark of PAH is dysfunction of the pulmonary artery endothelium, which leads to an imbalance of vasoactive mediators, including reduced nitric oxide (NO) and prostacyclin levels leading to vasoconstriction and impaired vasodilation [29]. Increased endothelin-1 (ET-1) levels promote PASMC proliferation and fibrosis [30]. Furthermore, studies have shown that oxidative stress and mitochondrial dysfunction play key roles in endothelial injury. Hydrogen sulfide (H2S) signaling has emerged as a novel modulator in PAH, inhibiting PASMC proliferation through endothelin receptor regulation [31].

### 3.2. Pulmonary Arterial Smooth Muscle Cell Proliferation and Resistance to Apoptosis

In PAH, PASMCs exhibit hyperproliferation and resistance to apoptosis, contributing to vascular remodeling and vessel occlusion. The key mechanisms include dysregulated BMPR2 signaling; loss-of-function mutations in bone morphogenetic protein receptor type 2 (BMPR2) lead to increased PASMC proliferation [32]. Long non-coding RNA (lncRNA) VELRP has been identified as a regulator of PASMC proliferation, promoting vascular remodeling [33]. Emerging studies suggest that restoring BMPR2 function or targeting PASMC survival pathways may offer novel therapeutic approaches.

### 3.3. Inflammatory and Immune Dysregulation

Chronic inflammation is increasingly recognized as a key driver of PAH progression. Elevated levels of interleukins (IL-6, IL-34), tumor necrosis factor-alpha (TNF-α), and C-reactive protein (CRP) have been observed in PAH patients [34]. Inflammation contributes to endothelial dysfunction as inflammatory cytokines promote vascular remodeling; fibrosis, as excessive collagen deposition, stiffens the pulmonary arteries; and macrophage and T-cell activation leads to sustained immune-mediated damage. Therapeutic strategies targeting immune modulation, such as JAK-STAT inhibitors, are currently being explored as potential PAH treatments [35,36].

### 3.4. Epigenetics and Genetic Modifications in PAH

Epigenetic mechanisms play a crucial role in PAH pathogenesis by regulating gene expression without altering DNA sequences. Aberrant DNA methylation and hypermethylation of anti-proliferative genes can lead to uncontrolled vascular cell growth [37]. Altered histone acetylation affects PASMC proliferation [38].

MicroRNAs (miRNAs) are small, non-coding RNAs that regulate gene expression post-transcriptionally, influencing cellular processes such as proliferation, apoptosis, and inflammation. In PAH, miR-204 downregulation is linked to increased PASMC proliferation and resistance to apoptosis, contributing to vascular remodeling. Restoring miR-204 levels has been proposed as a potential therapeutic strategy [39]. Epigenetic drugs, such as histone deacetylase, are also potential strategies to regulate gene expression [40].

### 3.5. Metabolic Dysregulation and Mitochondrial Dysfunction

PAH is associated with a shift in pulmonary vascular metabolism, resembling a cancer-like metabolic phenotype. Key metabolic changes include increased glycolysis, as PAH cells rely on anaerobic glycolysis instead of oxidative phosphorylation (Warburg effect) [41]. Reduced mitochondrial respiration leads to excessive ROS production [42]. PDE5-cGMP-PKG pathway dysregulation is also implicated in PASMC proliferation and right ventricular dysfunction [43]. Targeting metabolic reprogramming may provide novel therapeutic benefits.

**Figure 2:**
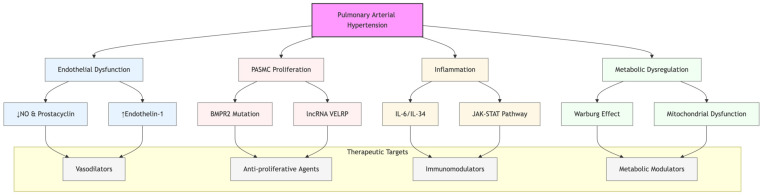
Pathophysiological mechanisms and therapeutic targets in pulmonary arterial hypertension (PAH). The diagram illustrates key pathways involved in PAH progression, including endothelial dysfunction, pulmonary arterial smooth muscle cell (PASMC) proliferation, inflammation, and metabolic dysregulation. Therapeutic strategies target these specific pathways through various mechanisms. NO: nitric oxide; BMPR2: bone morphogenetic protein receptor type 2; IL: interleukin; and JAK-STAT: Janus kinase-signal transducer and activator of transcription.

The pathophysiology of PAH is highly complex, involving endothelial dysfunction, vascular remodeling, chronic inflammation, epigenetic alterations, and metabolic reprogramming (Figure 2). Identifying key molecular drivers offers potential novel therapeutic targets, paving the way for precision medicine in PAH treatment.

## 4. Emerging Therapeutic Strategies for Pulmonary Arterial Hypertension (PAH)

Despite advancements in PAH pharmacotherapy, the current treatments primarily target vascular tone rather than disease progression. New therapeutic strategies focus on novel molecular pathways, immune modulation, metabolic reprogramming, and right ventricular (RV) support. This section explores emerging therapies, highlighting their mechanisms and potential clinical applications.

### 4.1. Targeting Pulmonary Vascular Remodeling

Pulmonary vascular remodeling is a key driver of PAH progression and is characterized by excessive PASMC proliferation and resistance to apoptosis. Recent therapeutic approaches target multiple pathways involved in this process through natural compounds, RNA-based interventions, and cell signaling modulators.

#### 4.1.1. Natural Compounds

Natural compounds have emerged as promising therapeutic agents for PAH, offering multiple mechanisms of action with potentially fewer side effects than synthetic drugs. 1,8-Cineole, a natural monoterpene, has demonstrated significant efficacy in reducing vascular remodeling by restoring intercellular communication and inhibiting angiogenesis. In preclinical studies, 1,8-Cineole treatment resulted in a reduction in pulmonary vascular resistance and improved right ventricular function [27].

Quercetin, a plant-derived flavonoid, acts through downregulation of the TGF-β1-Smad2/3 pathway. Studies have shown that quercetin treatment reduces pulmonary arterial pressure and decreases medial wall thickness in experimental PAH models [22]. The compound’s anti-inflammatory and antioxidant properties contribute to its therapeutic effects, making it a promising candidate for clinical development [39,44]. These findings suggest that targeting PASMC proliferation, endothelial dysfunction, and pro-angiogenic pathways may offer disease-modifying benefits.

#### 4.1.2. RNA-Based Interventions

RNA-based therapies represent a novel approach to targeting vascular remodeling in PAH. Long non-coding RNA VELRP has been identified as a crucial regulator of PASMC proliferation and vascular remodeling. Recent studies demonstrate that VELRP knockdown reduces pulmonary vascular resistance and improves survival in preclinical models [16]. The specificity of RNA targeting allows for precise modulation of disease-relevant pathways while minimizing off-target effects. This evidence suggests that lncRNA VELRP modulates PASMC proliferation and could serve as a therapeutic target [33,45].

MicroRNA-based interventions, particularly targeting miR-204, have shown promise in reversing the proliferative phenotype of PASMCs. The restoration of miR-204 levels reduces PASMC proliferation and enhances apoptotic responses, suggesting potential therapeutic applications [46].

#### 4.1.3. Cell Signaling Pathway Modulation

Targeting cellular signaling pathways offers opportunities for disease modification in PAH. BMPR2 signaling restoration represents a key therapeutic strategy, given its central role in pulmonary vascular homeostasis. Small-molecule BMPR2 activators have improved pulmonary hemodynamics in preclinical studies [47,48].

TGF-β pathway inhibition provides another promising approach, with selective inhibitors showing a reduction in pulmonary vascular resistance and improved right ventricular function in experimental models [49]. Combination approaches targeting multiple signaling pathways may offer enhanced therapeutic benefits.

**Figure 3:**
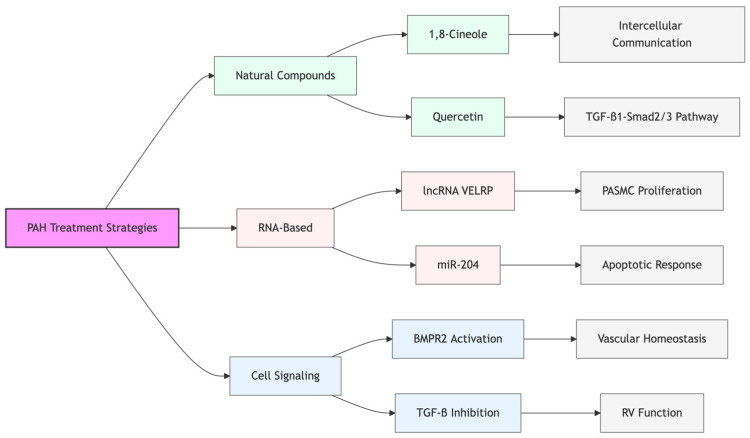
An overview of the emerging therapeutic strategies for PAH, showing three main approaches: natural compounds, RNA-based interventions, and cell signaling modulation. Each approach targets specific pathways and mechanisms involved in disease progression.

These diverse therapeutic strategies, as shown in Figure 3, target pulmonary vascular remodeling and show promise in preclinical studies and early clinical trials. Integration of these approaches with existing therapies may provide more effective treatment options for PAH patients.

### 4.2. Immunomodulatory and Anti-Inflammatory Therapies

Chronic inflammation plays a crucial role in PAH progression, and emerging therapies focus on immune modulation. Interleukin-6 (IL-6) Inhibitors: IL-6 blockade has shown promise in preclinical models, reducing pulmonary vascular inflammation and improving hemodynamics [50]. Janus kinase (JAK) inhibitors suppress cytokine-driven vascular remodeling and immune activation [36]. Elevated IL-34 levels correlate with PAH severity, and targeting this cytokine may offer new prognostic and therapeutic avenues [34]. Anti-inflammatory and immunomodulatory approaches could complement existing vasodilator therapies and improve long-term outcomes.

### 4.3. Metabolic Modulation in PAH

PAH is associated with dysregulated metabolism, mitochondrial dysfunction, and increased glycolysis (Warburg effect). Novel metabolic interventions include Multi-omics research that identified ETC dysfunction as a driver of PAH, with potential therapeutic targets in mitochondrial complex I/III [51]. PDE5-cGMP-PKG pathway modulation via Celastrol, a plant-derived compound, enhanced PDE5-cGMP-PKG signaling, reducing hypoxia-induced PAH [15]. Peroxisome Proliferator-Activated Receptor Gamma (PPAR-γ) agonists such as pioglitazone restore fatty acid oxidation and mitochondrial homeostasis, improving right ventricular function in PAH models [52]. Targeting metabolic pathways may shift the PAH phenotype from a proliferative to a quiescent state, reducing disease progression.

### 4.4. Novel Pharmacological Interventions

Several new pharmacological agents are in development, aiming to fill gaps in PAH management. Intravenous selexipag is a selective prostacyclin receptor agonist being evaluated for patients with progressive PAH who require parenteral therapy. Studies suggest that IV Selexipag may bridge gaps in oral treatment and improve exercise capacity [53]. Tyrosine kinase inhibitors (TKIs), agents that target vascular endothelial growth factor (VEGF) and platelet-derived growth factor (PDGF), are under investigation to prevent vascular proliferation [54]. Next-generation endothelin receptor antagonists (ERAs) are being explored for enhanced selectivity and reduced side effects [55]. These novel therapies aim to improve pulmonary vascular function, enhance cardiac performance, and prolong survival.

### 4.5. Right Ventricular-Directed Therapies

Right ventricular (RV) dysfunction is a critical determinant of prognosis in pulmonary arterial hypertension (PAH) and is the primary cause of death in affected patients [16]. Unlike the left ventricle, the RV is not well-adapted to sustained pressure overload, leading to progressive maladaptive remodeling [56], increased fibrosis [57], and eventual failure [58].

#### 4.5.1. Pathophysiology and Natural History of RV Dysfunction

The RV adapts to increased pulmonary vascular resistance through initial compensatory hypertrophy, which maintains cardiac output. However, as PAH progresses, chronic pressure overload leads to RV dilation and wall stress accumulation [59]; fibrotic remodeling and myocardial stiffening [60]; metabolic reprogramming, with a shift from fatty acid oxidation to glycolysis, resembling a failing heart [61]; and right atrial enlargement and eventual tricuspid regurgitation, exacerbating systemic venous congestion [62]. These maladaptive responses ultimately result in RV–pulmonary artery (RV-PA) uncoupling, where the RV is unable to effectively generate forward flow, leading to cardiogenic shock and death [63].

#### 4.5.2. Current Diagnostic Methods for Assessing RV Dysfunction

Early and accurate assessment of right ventricular (RV) dysfunction is essential in pulmonary arterial hypertension (PAH), given its strong prognostic implications. Echocardiography remains the most widely used non-invasive tool, with tricuspid annular plane systolic excursion (TAPSE) and RV fractional area change (FAC) serving as standard measures of RV function [64]. Advanced echocardiographic techniques, such as speckle-tracking echocardiography, allow for the assessment of RV longitudinal strain, providing a more sensitive evaluation of early RV impairment [65].

**Figure 4:**
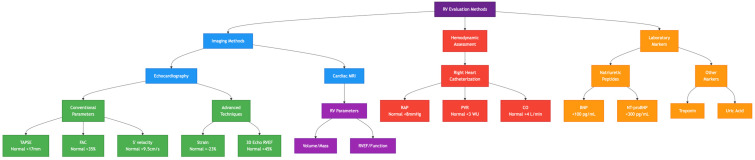
An overview of right ventricular evaluation methods.

Cardiac magnetic resonance (CMR) is considered the gold standard for evaluating RV function due to its superior accuracy in measuring RV ejection fraction (RVEF) and ventricular–vascular coupling [66]. CMR allows for a detailed assessment of RV structure, fibrosis, and contractility, making it a valuable tool for tracking disease progression and response to therapy. Right heart catheterization (RHC) provides direct hemodynamic measurements of RV function, including right atrial pressure, pulmonary artery pressure, and cardiac output [67]. One of the most predictive markers of RV failure obtained via RHC is the ratio of pulmonary arterial elastance (Ea) to RV end-systolic elastance (Ees), which serves as an indicator of RV-PA coupling [68]. Table 2 and Figure 4 display right ventricular evaluation methods and diagnostic modalities.

Emerging technologies are further advancing the field of RV assessment. Artificial intelligence-driven echocardiographic analysis is being explored to enhance the accuracy of RV strain detection, providing real-time insights into RV function, with one study of 7853 patients with right-sided heart catheterization demonstrating 82% accuracy and 88% sensitivity with their machine-learning model [69]. Additionally, non-invasive hemodynamic monitoring using wearable biosensors is an emerging approach that may allow for continuous tracking of RV performance in PAH patients [70]. These novel strategies showpromise for earlier detection of RV dysfunction, enabling more timely and personalized therapeutic interventions.

#### 4.5.3. Emerging Right Ventricle-Directed Therapies

Given that RV failure is the leading cause of death in PAH, there is an increasing focus on developing RV-targeted therapies that go beyond conventional vasodilator treatments. One promising area of research involves metabolic modulation, as PAH-induced RV failure is associated with a shift from oxidative phosphorylation to glycolysis, leading to inefficient energy production. Metabolic modulators such as peroxisome proliferator-activated receptor gamma (PPAR-γ) agonists, including pioglitazone, have shown potential in restoring fatty acid oxidation and improving RV function. PPAR-γ agonists and GLP-1 receptor agonists enhance RV metabolism and contractility [71]. Similarly, glucagon-like peptide-1 receptor (GLP-1R) agonists have been investigated for their ability to enhance RV mitochondrial efficiency and reduce metabolic stress [72].

Fibrotic remodeling is another critical factor in RV dysfunction, and therapies targeting excessive fibrosis are under investigation. Anti-fibrotic agents, such as pirfenidone and losartan, have shown promise in reducing RV stiffness and improving contractility. Additionally, inhibition of the transforming growth factor-beta (TGF-β) pathway is being explored as a potential strategy to limit pathological fibrosis and prevent further RV deterioration [73].

Another emerging therapeutic approach involves improving calcium handling within RV myocytes. Impaired calcium homeostasis is a key contributor to reduced RV contractility, and novel therapies aimed at enhancing sarco/endoplasmic reticulum Ca^2+^-ATPase 2a (SERCA2a) activity may help restore normal calcium cycling and improve RV performance [74]. Gene therapy approaches targeting calcium regulation are currently under preclinical investigation [75].

Reducing RV afterload is a crucial component of RV-directed therapy. Inhaled pulmonary vasodilators, such as nitric oxide and riociguat, have demonstrated efficacy in selectively lowering pulmonary arterial pressures without causing systemic hypotension [76]. In cases of severe RV failure, mechanical support devices, including RV assist devices, are being explored as potential bridge-to-recovery strategies [77]. By integrating novel imaging biomarkers with emerging RV-specific therapies, PAH management can evolve towards a more comprehensive RV-centered approach, potentially improving both functional capacity and long-term survival in affected patients. Future research should focus on validating these targeted interventions in clinical trials to determine their efficacy in modifying the disease course and preventing RV failure. Table 3 summarizes emerging right ventricular-directed therapies.

## 5. Challenges in PAH Research

Despite recent advancements, PAH remains a high-mortality disease with unresolved research gaps:

### 5.1. Heterogeneity of PAH Etiology

PAH encompasses multiple subtypes (idiopathic, heritable, and associated with systemic diseases), each with unique molecular drivers. Personalized therapeutic approaches are needed to tailor treatments based on disease subtypes [78].

### 5.2. Lack of Early Diagnostic Biomarkers

Current PAH diagnosis relies on invasive right heart catheterization (RHC). Research is exploring non-invasive biomarkers such as circulating microRNAs, exosomal signatures, and epigenetic markers [79].

### 5.3. Limited Translational Success

Many preclinical drug candidates fail in clinical trials due to differences between animal models and human disease. Better in vitro models (e.g., human-induced pluripotent stem cells, organ-on-chip technologies) may improve predictive validity [80].

### 5.4. Right Ventricular Dysfunction Is Understudied

Most PAH research focuses on pulmonary vasculature, neglecting right ventricular failure, which is the main cause of death. RV-targeted therapies need further investigation [81].

## 6. Future Research Directions

### 6.1. Precision Medicine Approaches

PAH is increasingly recognized as a disease with diverse genetic and molecular drivers. Personalized therapy based on genomic, proteomic, and metabolomic profiling is a promising avenue. Single-cell RNA sequencing (scRNA-seq) is identifying unique transcriptional changes in endothelial and PASMCs [82]. Multi-omics integration (transcriptomics, metabolomics) is uncovering novel therapeutic targets [83].

### 6.2. Novel Drug Discovery and Repurposing

#### 6.2.1. Gene Therapy and RNA-Based Interventions

Gene editing (CRISPR-based approaches) could restore BMPR2 function in hereditary PAH [84]. Antisense oligonucleotides (ASOs) and small interfering RNAs (siRNAs) are being explored to regulate disease-driving genes [85].

#### 6.2.2. Stem Cell and Extracellular Vesicle (EV) Therapy

Mesenchymal stromal cell (MSC)-derived extracellular vesicles show anti-inflammatory and regenerative properties in preclinical PAH models [86]. MSCs may help repair endothelial dysfunction and reverse vascular remodeling [87].

### 6.3. Advanced Imaging and Non-Invasive Diagnostics

Early and accurate PAH diagnosis remains challenging. Future diagnostics will focus on artificial intelligence (AI)-driven imaging. AI-enhanced echocardiography and machine-learning algorithms can improve PAH detection and prognosis prediction [69]. Circulating biomarkers such as exosomal long non-coding RNAs (lncRNAs) and microRNAs (e.g., miR-204) may serve as liquid biopsy tools [88]. Non-invasive pressure monitoring via emerging technologies, such as wearable biosensors, could allow continuous pulmonary pressure monitoring [89].

### 6.4. Overcoming Barriers to Clinical Translation

For novel PAH therapies to reach patients, research must address trial design and patient recruitment challenges. PAH is a rare disease, making large-scale clinical trials difficult [90]. International PAH patient registries can facilitate recruitment and improve statistical power. The development of 3D lung organoids, humanized animal models, and multi-cellular PAH models can help bridge the gap between preclinical models and human disease [91].

## 7. Conclusions

PAH remains a progressive and life-threatening disease, necessitating a paradigm shift in treatment approaches beyond conventional vasodilatory therapies. The integration of molecular-targeted treatments, AI-driven diagnostics, and personalized medicine marks a transformative phase in PAH management. However, critical challenges remain, including early disease detection, right ventricular failure interventions, and the clinical translation of novel therapies. Future research efforts should prioritize large-scale clinical trials, real-world validation of AI-assisted diagnostics, and patient-specific therapeutic strategies. By addressing these challenges, PAH treatment can evolve from symptomatic management to disease modification, ultimately improving survival and quality of life for affected individuals.

### 7.1. Key Takeaways from This Review

#### 7.1.1. Pathophysiology and Molecular Mechanisms

PAH is driven by endothelial dysfunction, PASMC hyperproliferation, inflammation, metabolic dysregulation, and epigenetic modifications [92]. BMPR2 mutations, JAK-STAT signaling, and mitochondrial dysfunction are emerging therapeutic targets [93].

#### 7.1.2. Emerging Therapies

Novel vascular remodeling inhibitors (e.g., 1,8-Cineole, quercetin) show promise in experimental models [94]. Metabolic modulators, immunotherapy approaches, and right ventricular-directed therapies represent new therapeutic frontiers. Gene-based interventions, RNA therapeutics, and stem cell-derived extracellular vesicles (EVs) hold potential for disease modification [95].

#### 7.1.3. Future Directions and Research Challenges

Early diagnosis and personalized medicine remain top priorities. Artificial intelligence (AI)-driven diagnostics and multi-omics approaches will enhance PAH phenotyping and therapeutic targeting [96]. Bridging the translational gap between preclinical research and human trials is crucial for effective drug development.

### 7.2. Final Remarks

The future of PAH research and therapy lies in integrating molecular insights with precision medicine, optimizing targeted interventions, and ensuring early, non-invasive diagnostics. The next generation of PAH therapies aims not only to prolong survival but also to improve patient quality of life through disease-modifying interventions.

## Figures and Tables

**Table 1:** Classification of pulmonary hypertension.

PH Group	Etiology	Pathophysiology	Key Diagnostic Features	Treatment Approach
Group 1: PAH	Idiopathic, heritable, drug-induced, connective tissue disease-associated	Endothelial dysfunction, vascular remodeling, increased pulmonary vascular resistance	Right heart catheterization: Mean PAP > 25 mmHg, PCWP < 15 mmHg	Vasodilators (prostacyclins, PDE5 inhibitors, ERAs), supportive care
Group 2: PH due to left heart disease	Heart failure (HFrEF, HFpEF), valvular disease	Passive pulmonary congestion, elevated left atrial pressures	PCWP > 15 mmHg, echocardiography showing left-sided dysfunction	Treat underlying heart disease, avoid PAH-specific vasodilators
Group 3: PH due to lung disease/hypoxia	COPD, interstitial lung disease, sleep apnea	Hypoxic vasoconstriction, vascular remodeling	Pulmonary function tests, high-resolution CT, V/Q scan	Oxygen therapy, treat underlying lung disease
Group 4: CTEPH	Chronic thromboembolic disease, recurrent PE	Pulmonary artery obstruction, secondary vascular remodeling	V/Q scan, CT angiography, pulmonary angiography	Pulmonary endarterectomy, riociguat, anticoagulation
Group 5: PH with unclear/multifactorial mechanisms	Sarcoidosis, hematologic disorders, metabolic diseases	Mixed pathophysiological mechanisms, unclear primary drivers	Variable diagnostic findings	Disease-specific management

**Table 2:** Diagnostic modalities for right ventricular dysfunction in PAH.

Modality	Key Parameters	Advantages	Limitations
Echocardiography	TAPSE, FAC, RV longitudinal strain	Non-invasive, widely available	Limited sensitivity for early dysfunction
Cardiac MRI	RV ejection fraction, fibrosis assessment	Gold standard for RV function, precise imaging	Expensive, less accessible
Right Heart Catheterization	RV pressures, RV-PA coupling (Ea/Ees ratio)	Direct hemodynamic measurements, high prognostic value	Invasive, procedural risks
AI-Driven Echocardiography	Automated RV strain analysis	Emerging technology, improved accuracy	Requires further validation
Wearable Biosensors	Continuous hemodynamic monitoring	Potential for early detection	Experimental stage

**Table 3:** Emerging right ventricular-directed therapies.

Therapeutic Strategy	Mechanism of Action	Potential Benefits	Current Status
Metabolic Modulation (PPAR-γ agonists, GLP-1R agonists)	Restores RV fatty acid oxidation and mitochondrial function	Improves RV efficiency, delays dysfunction	Preclinical/early clinical trials
Fibrosis Inhibition (Pirfenidone, TGF-β inhibitors)	Reduces RV collagen deposition and stiffness	Prevents RV remodeling	Preclinical studies
Calcium Handling Modulation (SERCA2a enhancement)	Restores calcium cycling in RV myocytes	Improves contractility	Experimental
RV Afterload Reduction (inhaled vasodilators, riociguat)	Lowers pulmonary pressures selectively	Reduces RV workload, improves function	Clinical use
Mechanical Support (RV assist devices)	Provides temporary RV circulatory support	Bridge to recovery or transplant	Investigational

## Data Availability

Data are available upon reasonable request to authors.

## Abbreviations

The following abbreviations are used in this manuscript:

PAH	Pulmonary Arterial Hypertension
RV	Right Ventricle
PVR	Pulmonary Vascular Resistance
PASMCs	Pulmonary Arterial Smooth Muscle Cells
BMPR2	Bone Morphogenetic Protein Receptor Type 2
ET-1	Endothelin-1
NO	Nitric Oxide
Jak-Stat	Janus-Kinase-Signal Transducer and Activator of Transcription
HDAC	Histone Deacetylase
TGF-β	Transforming Growth Factor-Beta
miRNA	MicroRNA
lncRNA	Long Non-Coding RNA
PDE5	Phosphodiesterase Type 5
cGMP	Cyclic Guanosine Monophosphate
PKG	Protein Kinase G
EVs	Extracellular Vesicles
MSC	Mesenchymal Stromal Cells
AI	Artificial Intelligence
scRNA-seq	Single-Cell RNA Sequencing
RHC	Right Heath Catheterization
